# Cell surface CD11c as a neutrophil aging marker molecule

**DOI:** 10.1002/2211-5463.70332

**Published:** 2026-08-31

**Authors:** Sophia Koutsogiannaki, Fahd Alhamdan, Erik Malm, Marie Bermudez, Samuel Y. Kim, Koichi Yuki

**Affiliations:** ^1^ Department of Anesthesiology, Critical Care and Pain Medicine Boston Children's Hospital Massachusetts USA; ^2^ Department of Anaesthesia Harvard Medical School Boston Massachusetts USA; ^3^ Department of Immunology Harvard Medical School Boston Massachusetts USA; ^4^ Broad Institute of MIT and Harvard Cambridge Massachusetts USA

**Keywords:** CD11c, cell surface, Integrin, neutrophils

## Abstract

Neutrophils exhibit substantial functional heterogeneity shaped by both developmental stage and cellular maturation, but their mechanisms remain incompletely defined. CD11c (ITGAX) is traditionally considered as a cell surface marker of dendritic cells. We recently reported that it is highly expressed intracellularly in neutrophils and contributes to their maturation; however, its role at the cell surface is unclear. Here, we showed that surface CD11c expression level was heterogeneous across neutrophils, enabling classification into CD11c^hi^ and CD11c^−/lo^ populations. CD11c^hi^ neutrophils display enhanced phagocytic capacity and features of neutrophil aging, including increased CXCR4 and reduced CD62L expression. Bulk RNA sequencing across the three pediatric age groups revealed that differentially expressed genes (DEGs) between CD11c^hi^ and CD11c^−/lo^ neutrophils were observed most in infants followed by preschool age children. Infant neutrophils exhibited reduced phagocytic capacity and distinct gene expression patterns compared to older children. Functional interrogation of candidate genes (ANXA2, COL6A3, DCN) demonstrated their role in regulating phagocytosis without affecting reactive oxygen species production. Machine learning analysis further identified age‐dependent ontology of the DEGs associated with cell surface CD11c expression, including pathways related to adhesion, extracellular matrix interactions, stress responses, and metabolic regulation. Integrative network analysis positioned CD11c linked to cytoskeletal remodeling and phagocytosis. Collectively, these findings support a model in which developmental age establishes the baseline transcriptional landscape of neutrophils, while CD11c serves as a marker of neutrophil aging rather than acting as a primary driver of transcriptional reprogramming.

## Graphical abstract

Cell surface CD11c^hi^ neutrophils were more aged and had better phagocytic function than CD11c^−/lo^ neutrophils. Transcriptomic analysis of CD11c^hi^ neutrophils and CD11c^−/lo^ neutrophils in pediatric population showed that the most difference was seen in infants. Among the differentially expressed genes, Anxa2, Col6a3, and Dcn were involved in phagocytosis.

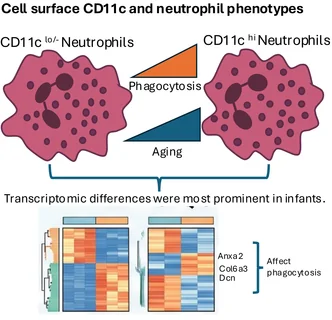

**Abbreviations**
ANOVA: one‐way analysis of variance
DEG: Differentially expressed gene
FACS: fluorescence‐activated cell sorting
FDR: false discovery rate
HIV: human immunodeficiency virus
IPA: ingenuity pathway analysis
MPO: myeloperoxidase
NETs: neutrophil extracellular traps
PC1: principal component 1
PCA: principal component analysis
PCR: polymerase chain reaction
PFA: paraformaldehyde
PMA: phorbol‐12‐myristate‐13‐acetate
RNP: ribonucleoprotein
ROS: reactive oxygen species
TNF: tumor necrosis factor

While CD11c has been primarily used as a cell surface marker for dendritic cells in the immunology field for many years, its functional role *in vivo* remains incompletely defined and relatively underexplored. CD11c belongs to the adhesion molecule family of integrin and couples with CD18 to form a heterodimeric receptor complex. This integrin complex has been shown to bind to complement component iC3b *in vitro* [1], suggesting that CD11c may have an important functional role beyond its use as a phenotypic marker.

Previously, we demonstrated that CD11c was highly expressed in the intracellular space of neutrophils in a range of developmental stages, contributing to their maturation in the bone marrow [2]. Complementary evidence from proteomics and gradient‐based granule separation studies showed that intracellular CD11c was localized in the granules of neutrophils [3, 4]. We also noted that CD11c was expressed on the cell surface of neutrophils, although its expression level was significantly lower than intracellularly. However, it is not yet determined if cell surface CD11c has any functional implication in neutrophils. In the context of systemic inflammation, higher CD11c cell surface expression on neutrophils was considered a significant risk factor for sepsis in adults [5], suggesting its potential clinical/functional relevance.

Neutrophils are generated in the bone marrow and released into circulation as terminally differentiated effector cells. As neutrophils are known to have a short half‐life at the order of 4 ~ 19 hours [6, 7, 8, 9], their lifespan is tightly regulated, and at the end of their life cycle, they either undergo constitutive apoptosis or home back to the bone marrow for clearance via phagocytosis. Functionally, a subset of integrins plays a critical role in neutrophil adhesion and activation. Given the broad range of neutrophil effector functions, including adhesion, migration, phagocytosis, and inflammatory signaling, it is important to determine whether cell surface CD11c on neutrophils also plays a unique functional role, thereby further clarifying the relatively underexplored role of CD11c in innate immunity. To address these knowledge gaps, we designed the present study to define the functional role of cell surface CD11c in neutrophils. Specifically, we compared neutrophil functional properties by sorting CD11c^−/lo^ and CD11c^hi^ populations, enabling us to directly assess whether neutrophils with different CD11c expression are associated with distinct neutrophil phenotypes. Prior work from our group demonstrated that pediatric leukocytes undergo significant transcriptomic changes along with their developmental growth [10]. Thus, we also aimed to evaluate the transcriptomic relationship with cell surface CD11c in different age groups. Defining the functional role of CD11c in neutrophils is important, as it challenges the traditional view of CD11c as solely a phenotypic marker and instead suggests a role in regulating innate immune function.

## Methods

### Human subject enrollment

The study was approved by the Institutional Review Board of Boston Children's Hospital (IRB‐P00031930), and written informed consent was obtained from parents or legal guardians; assent was also obtained from patients when appropriate. All study procedures were conducted in accordance with institutional guidelines and ethical standards for human subject research. Subjects were prospectively enrolled between May 2021 and January 2023. All procedures adhered to the Declaration of Helsinki, and the study was registered at ClinicalTrials.gov (NCT04103268). Eligible participants were aged 1 month to 18 years, representing otherwise healthy pediatric patients undergoing elective surgical procedures or healthy adult volunteers. Exclusion criteria included congenital heart diseases, malignancy, autoimmune disorders, organ transplantation, human immunodeficiency virus (HIV) infection, or corticosteroid therapy to minimize potential confounding effects on immune cell functions and inflammatory responses.

### Flow cytometry study of neutrophils

A total of 1 mL of peripheral blood was collected in heparin‐coated tubes and processed immediately to preserve cellular integrity. Neutrophils were isolated and sorted into CD11c^hi^ and CD11c^−/lo^ populations by fluorescence‐activated cell sorting (FACS) based on surface CD11b^+^, CD15^+^ gating using CD11c‐FITC (clone: Bu15), CD11b‐PE (clone: CBRM1/5), and CD15‐APC (clone: W6D3) antibodies (all from BioLegend, San Diego, CA, USA). Sorted cell pellets were rapidly collected and immediately stored at −80 °C to preserve RNA integrity and were subsequently subjected to bulk RNA sequencing analysis to assess transcriptional differences between CD11c‐defined neutrophil subsets.

### Neutrophil extracellular traps (NETs) formation

Human neutrophils (2.5 × 10^5^ in 200 μL) in complete RPMI 1640 with 10 mm HEPES and 50 μm 2‐ME were allowed to adhere to eight‐well glass chamber slides (Thermo Fisher Scientific) for 1 h at 37 °C followed by phorbol‐12‐myristate‐13‐acetate (PMA) (50 nm) incubation for 4 h. To examine NETs, the cells at the termination of NET induction were washed once with warm PBS, and then, Sytox green (0.1 μm) was added. The NETs were imaged using a fluorescence microscope, Zeiss Axiovert (Carl Zeiss; Thornwood, NY). We also fixed cells with 4% paraformaldehyde (PFA) and then stained with antimyeloperoxidase (MPO) antibody (BD Biosciences, Franklin Lakes, NJ), DAPI, and Sytox green.

### Reactive oxygen species (ROS)

Human neutrophils (2 × 10^5^ in 200 μL) were cultured in complete RPMI 1640 at 37 °C for 30 min. Dihydrorhodamine‐123 (1 μm; Sigma‐Aldrich, St. Louis, MO) was added for 5 min at 37 °C. Neutrophils were washed once. PMA (100 nm) was added, and the cells were incubated for an additional 15 min at 37 °C. After one wash, the cells were resuspended in cold PBS with 1% FBS for detection of ROS‐induced rhodamine‐123 on a FACSCanto (BD Biosciences). Regarding ROS experiment using HL‐60‐derived neutrophils, they were cultured in RPMI 1640 at 37 °C for 30 min in the presence of PMA (100 nm) [11].

### Phagocytosis

The neutrophil phagocytosis was done using the Phagotest Kit (Glycotope Biotechnology; Heidelberg, Germany). Human neutrophils or HL‐60 derived neutrophils were cultured in complete RPMI 1640 on ice for 10 min, followed by the addition of FITC‐*E. Coli*. Then, the neutrophil suspension was kept on ice as negative control or was placed into 37 °C water bath for 10 min. At the end of incubation, cells were transferred back on ice, quenched, and washed. Cells were suspended in PBS with 1% paraformaldehyde (PFA) and measured by FACSCanto (BD Biosciences) and analyzed by FlowJo software (Tree Star, Ashland, OR).

### RNA sequencing experiments

Freshly sorted CD11c^hi^ and CD11c^−/lo^ neutrophils were subjected to RNA extraction using the Qiagen RNeasy Plus Mini Kit according to the manufacturer's protocol. RNA quantity was assessed using the Qubit 2 Fluorometer (Life Technologies, Carlsbad, CA), and RNA integrity was evaluated with the Agilent TapeStation (Agilent Technologies, Palo Alto, CA). Full‐length cDNA synthesis and amplification were performed using the SMART‐Seq v4 Ultra Low Input Kit (Clontech, Mountain View, CA). Sequencing libraries were generated using the Illumina Nextera XT library preparation kit, which employs transposase‐mediated fragmentation and adaptor ligation, followed by limited‐cycle PCR for library enrichment and indexing. Final libraries were quantified and quality‐checked using Qubit and TapeStation prior to sequencing. Libraries were multiplexed, pooled, and sequenced on an Illumina HiSeq platform using a paired‐end 2 × 150 bp configuration. Raw sequencing reads underwent quality control and preprocessing, including adapter trimming and removal of low‐quality bases using Trimmomatic (v0.36). Processed reads were aligned to the human reference genome using STAR aligner (v2.5.2b), and only uniquely mapped reads within annotated exon regions were retained for downstream analysis. Sequencing quality was assessed using standard quality‐control metrics. Samples achieved a sequencing depth ranging from 5 to 9 million reads per sample. The average Phred quality score of the sequenced reads exceeded 34, indicating high base‐calling accuracy. GC content ranged from 44% to 48% across samples, consistent with expected transcriptome composition. Alignment of reads to the reference genome yielded overall mapping rates of 86–90%, with 80–83% of reads mapping uniquely to a single genomic location. All samples met the established quality‐control criteria and were included in downstream analyses. Gene‐level count matrices were generated and analyzed using DESeq2. To account for potential technical variation, batch information was included as a covariate in the design formula when applicable (design = ~ batch + condition). Differential expression testing was then performed using the DESeq2 statistical framework. Differential gene expression between groups was assessed using the Wald test, with genes defined as differentially expressed based on an adjusted *P*‐value < 0.05 and an absolute log_2_ fold change > 1. Functional enrichment and pathway analysis were performed using ingenuity pathway analysis (IPA; Qiagen), clusterProfiler package in R (V 4.6.2), and AI‐based tool GeneAgent (V 1.0:20250418) [12]. RNA sequencing data were deposited in GEO (GSE336366).

### HL‐60 derived neutrophils and CRISPR/Cas9 gene editing

CRISPR/Cas9‐mediated gene editing to delete ANXA2, COL6A3, DCN expression was done in HL‐60 cells (obtained from ATCC, Manassas, Virginia) as follows. HL‐60 cells were cultured at 37 °C in RPMI1640 supplemented with 10% FBS, 1% penicillin/streptomycin. Confluency was maintained between 3 × 10 [5]–1.5 × 10^6^·mL^−1^ to ensure optimal growth conditions. Electroporation was performed one day after passaging when cells were in a logarithmic growth phase, using the Lonza 4D Nucleofector system with 20 μL Nucleocuvette strips as previously described [13, 14]. Briefly, ribonucleoprotein (RNP) complexes were made by combining 100 pmol Cas9 (IDT; Newark, NJ) with 100 pmol modified sgRNA (Synthego; Redwood City, CA) targeting ANXA2 (CCAAATCACCGTCTCCAGG), COL6A3 (TGTTGTTTGATCCATCAATA), DCN (AAGCAGAAGGAGGATGATAG), and incubating at room temperature for 15 min. A nontargeting control was included using sgRNA directed against the AAVS1 locus (GGGGCCACTAGGGACAGGAT). For nucleofection, 2 × 10 [5]–4 × 10^5^ HL‐60 cells were resuspended in 20 μL SF cell line solution (Lonza; Basel, Switzerland), mixed with RNP complexes, and electroporated using program EN‐138 per manufacturer's recommendations. Following nucleofection, cells were returned to complete RPMI 1640 medium and cultured under standard conditions. Editing efficiency was assessed 48‐h postelectroporation by polymerase chain reaction (PCR). First, genomic DNA was extracted using the DNeasy kit (Qiagen; Hilden, Germany) according to the manufacturer's instructions. Target loci were amplified by using Platinum II Hotstart Mastermix (Thermo Fisher Scientific) and editing efficiency was quantified by Sanger sequencing. Edited allele frequencies were analyzed using the ICE (Inference of CRISPR Edits) tool [15]. In addition, the degree of protein deletion was assessed by flow cytometry analysis. Intracellular expression of Annexin A2, COL6A3 and DCN was examined using Annexin A2 antibody (Cell Signaling, Danvers, MA), collagen type 6A3 antibody (Sigma‐Aldrich) and Decorin antibody (Abcam, Waltham, MA). In briefly, following fixation, cells were subjected to permeabilization using permeabilization buffer followed by antibody staining. HL‐60 differentiation into neutrophil‐like cells was induced with 1 μm all‐trans retinoic acid (ATRA, Sigma‐Aldrich) and 1.25% DMSO for 4 days as we previously performed [2].

### Statistical analysis

The data are shown as means ± SDs. Statistical analyses were performed using GraphPad Prism (version 11; GraphPad Software, San Diego, CA) and R (version 4.5.3). Comparisons between two groups were conducted using an unpaired two‐tailed Student's *t*‐test. For comparisons involving more than two groups, one‐way analysis of variance (ANOVA) was applied, followed by Bonferroni's *post hoc* test to correct for multiple comparisons. A *P*‐value of < 0.05 was considered statistically significant. For high‐dimensional data analyses, including RNA sequencing, differential expression analysis was performed using DESeq2, with statistical significance defined as an adjusted *P*‐value (false discovery rate, FDR) < 0.05.

## Results

### CD11c expression defines a functionally distinct neutrophil subset with enhanced phagocytic capacity

**Fig. 1:**
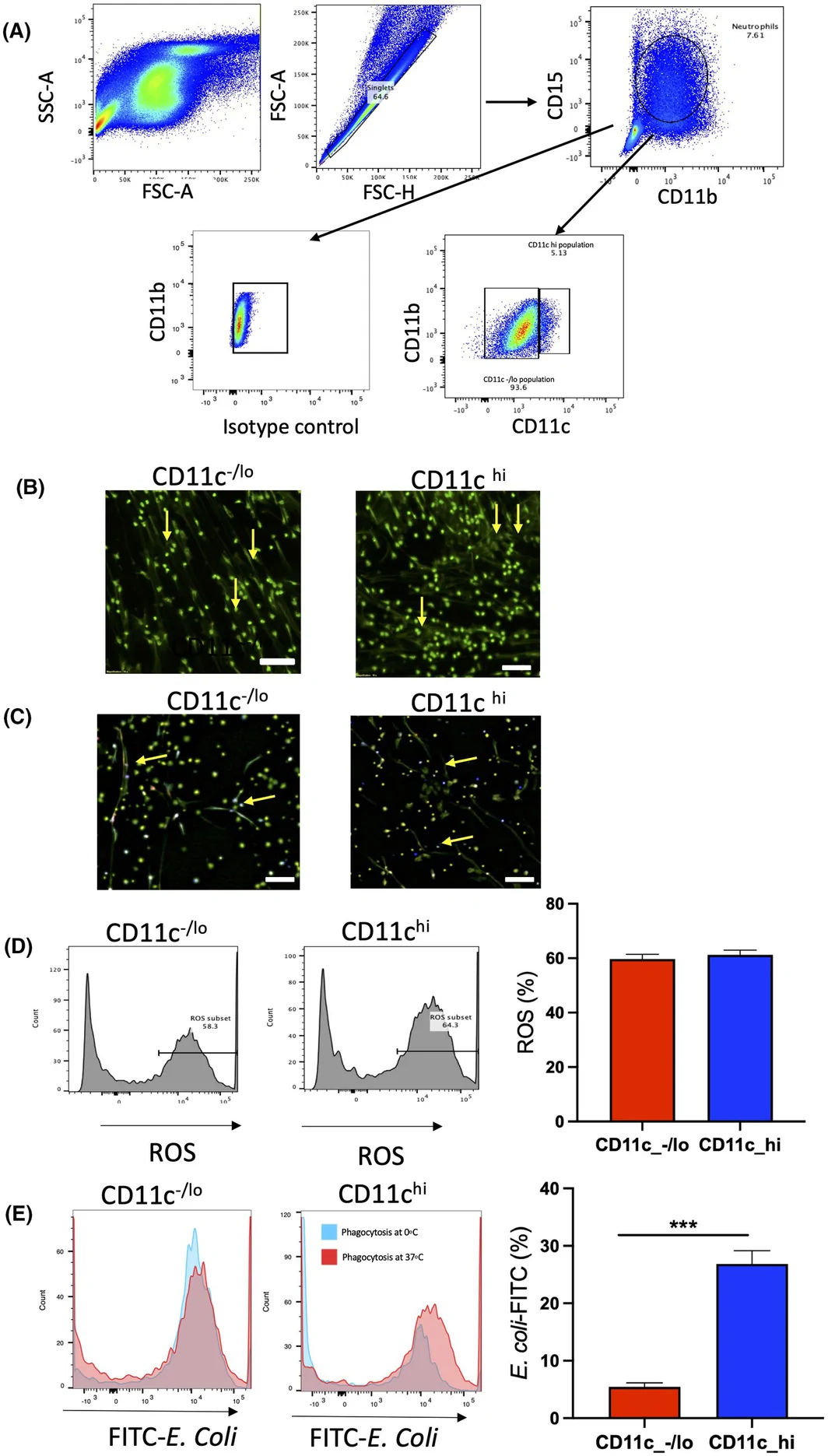
The comparison of effector functions between cell surface CD11c^hi^ and CD11c^−/lo^ neutrophils. CD11c^hi^ and CD11c^−/lo^ neutrophils were sorted for effector function assays. (A) Gating strategy of CD11c^−/lo^ and CD11c^hi^ neutrophils. (b, c) Neutrophil extracellular traps (NETs) formation was examined under PMA (100 ng/mL) stimulation. A representative image was shown from the three independent experiments. White bar represents 100 μm. (B) NETosis was evaluated by Sytox green. (C) Myeloperoxidase (MPO) (red), sytox green (green), and DAPI (blue) were used to assess NETs formation. (D) Reactive oxygen species (ROS) was examined using DHR123 under PMA (100 ng·mL^−1^) stimulation. Left panel: The representative histogram plot. Right panel: ROS analysis of three independent experiments shown as mean ± S.E. Student *t*‐test was performed. No statistical difference was observed. (E) Phagocytosis of *E. coli*‐FITC was evaluated. Left panel: The representative histogram plot. Right panel: Phagocytosis analysis of three independent experiments shown as mean ± S.E. Student *t*‐test was performed. ***P < 0.001.

To investigate whether cell surface CD11c is associated with functional heterogeneity in neutrophils, we sorted CD11c^hi^ and CD11c^−/lo^ neutrophil populations from healthy volunteers (Fig. 1A) and systematically assessed their effector functions. Previous studies have suggested that cell surface CD11c acts as a fibrinogen receptor on TNF‐α stimulated neutrophils [16]; however, the broader role of cell surface CD11c in neutrophil biology remains unclear.

Using healthy adult volunteers' blood‐derived neutrophils, we first evaluated NETs formation and observed that both CD11c^hi^ and CD11c^−/lo^ neutrophils formed NETs, indicating that cell surface CD11c expression did not significantly influence this effector pathway (Fig. 1B,C). The assessment of ROS production revealed no significant differences between the two populations (Fig. 1D), suggesting that oxidative burst capacity was not dependent on cell surface CD11c expression levels. As PMA‐mediated ROS and NETs formation use the same overlapping pathway with ROS being a critical intermediate step [17], these results are consistent. In contrast, the functional assessment of phagocytosis demonstrated that CD11c^hi^ neutrophils exhibited significantly enhanced phagocytic activity compared to CD11c^−/lo^ neutrophils (Fig. 1E).

### CD11c expression correlates with CD11b but does not independently mediate neutrophil phagocytosis

**Fig. 2:**
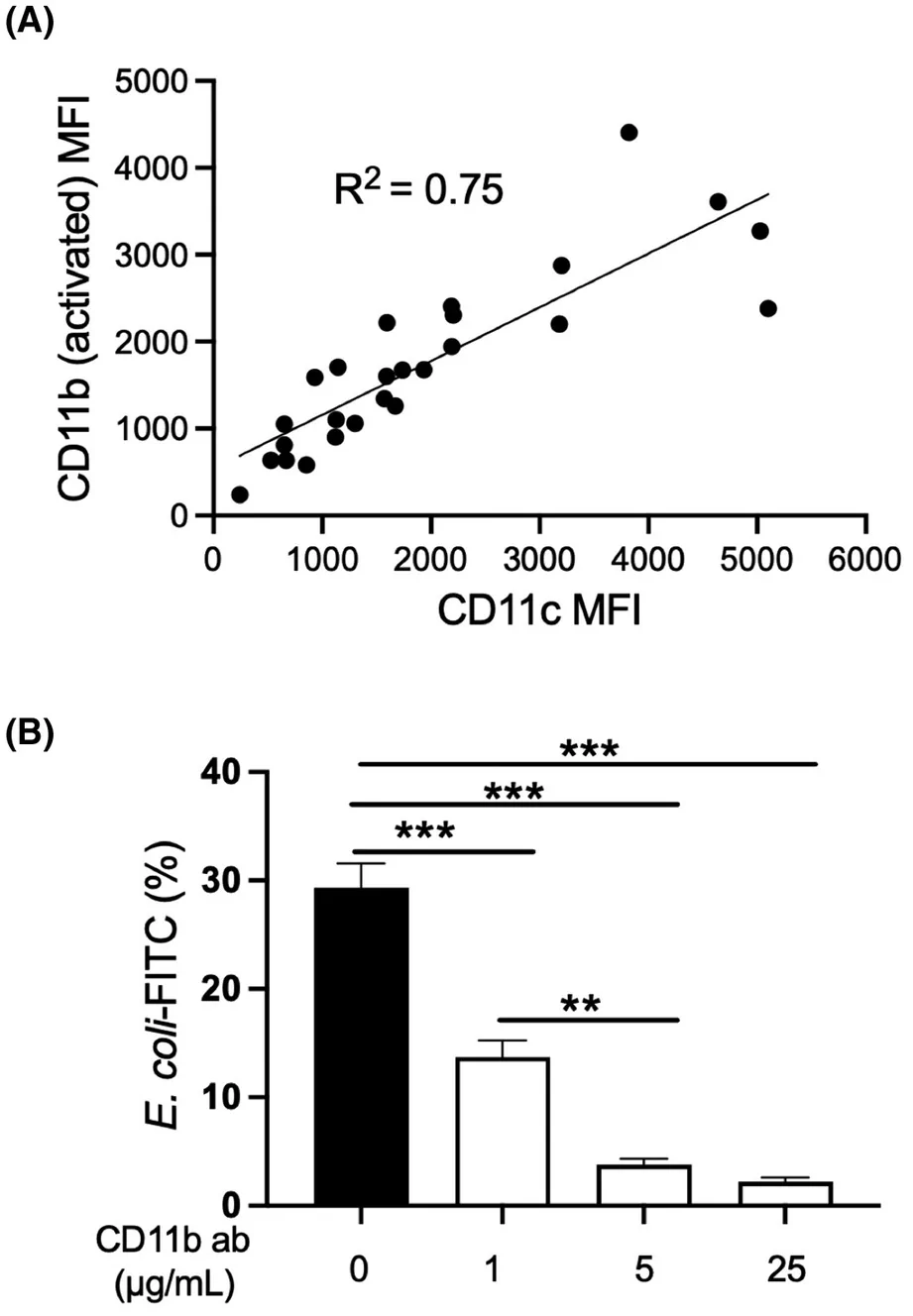
The relationship between cell surface CD11b and CD11c in neutrophils. (A). The correlation between CD11b and CD11c cell surface expression was determined using linear regression analysis was tested by combining data points from 6 independent donors. From each donor, 4–5 locations on CD11b vs CD11c plot were chosen to obtain average CD11b and CD11c values of the spot. *R* ^2^ values were shown. CD11b (activated) indicates activation‐sensitive antibody. (B) Neutrophil phagocytosis was tested with or without CD11b antibody at three different concentrations (1, 5, and 25 μg·mL^−1^). Data were shown as mean ± S.E. of three independent experiments. One‐way ANOVA with Bonferroni *post hoc* analysis was done. **P < 0.01. ***P < 0.001.

Given that CD11b is a well‐established integrin receptor mediating neutrophil phagocytosis [18], we next examined the relationship between cell surface CD11c and CD11b expression to determine whether the enhanced phagocytic capacity observed in CD11c^hi^ neutrophils would be directly attributable to CD11c or reflects co‐expression with CD11b. Flow cytometric analysis revealed a strong linear correlation between CD11c and CD11b expression on the neutrophil surface (Fig. 2A). To directly assess the functional contribution of CD11b to phagocytosis, we performed blocking experiments using a CD11b‐specific blocking antibody. Inhibition of CD11b completely abrogated phagocytic activity (Fig. 2B), demonstrating that CD11b is the primary receptor mediating this process under these conditions. In another word, an enhanced phagocytosis in CD11c^hi^ neutrophils was mediated primarily by CD11b co‐expression.

Taken together, these findings indicate that while cell surface CD11c expression is associated with enhanced phagocytic capacity, it does not independently drive phagocytosis. Instead, CD11c may serve as a marker of a neutrophil subset with increased CD11b expression and functional competence. This distinction highlights the importance of differentiating between markers of activation and direct mediators of effector function in neutrophil biology.

### CD11c expression identifies an aged neutrophil phenotype defined by canonical maturation markers

Given the established role of CD11b as a marker of neutrophil aging and activation [19], and the observed linear relationship between CD11b and CD11c expression (Fig. 2A), we hypothesized that cell surface CD11c may similarly reflect neutrophil aging status. To test this, we examined the expression of established aging‐associated markers, including CXCR4, which is upregulated in aged neutrophils that home back to the bone marrow, and CD62L (L‐selectin), which is shed during neutrophil activation and aging [20].

**Fig. 3:**
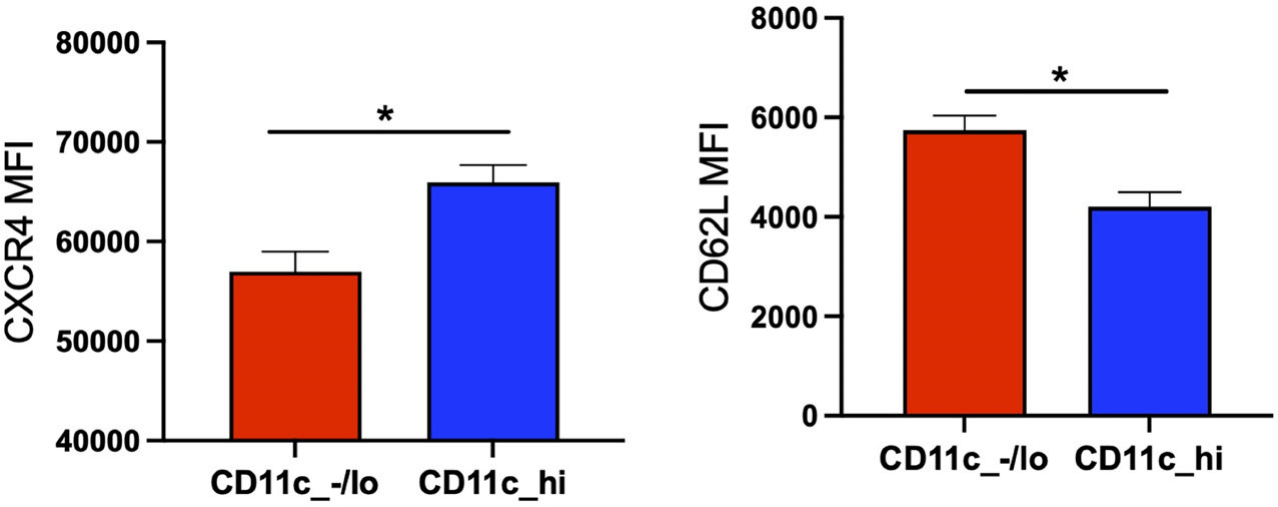
Relationship between cell surface CD11c expression and neutrophil aging. Cell surface CD11c^hi^ and CD11c^−/lo^ neutrophils were examined for their expression of neutrophil aging markers CXCR4 and CD62L. Data were shown as mean ± S.E. of three independent experiments. *P < 0.05.

We found that CD11c^hi^ neutrophils exhibited significantly higher CXCR4 expression and reduced CD62L expression compared to CD11c^−/lo^ neutrophils (Fig. 3), a phenotype consistent with an aged or terminally differentiated neutrophil subset. These findings support the concept that CD11c expression stratifies neutrophils along a maturation or aging axis and suggest that CD11c serves as an additional marker of neutrophil aging.

Together, these data indicate that CD11c^hi^ neutrophils represent a more mature and functionally distinct population, providing a framework to interpret their enhanced phagocytic capacity observed in earlier analyses.

### CD11c‐defined neutrophil subsets exhibit minimal global transcriptomic differences within age groups

To determine whether the functional differences associated with CD11c expression are reflected at the transcriptional level, we performed bulk RNA sequencing on sorted CD11c^hi^ and CD11c^−/lo^ neutrophils. Given well‐established age‐dependent variability in immune cell function, particularly in pediatric population [21], we stratified the cohort into three developmental groups to account for differences in immune state across the age groups. The demographics of the cohort were shown in Table 1.

**Table 1:** Demographics of healthy pediatric cohorts enrolled for the study.

Infant		Preschool		School	
Age (months)	Sex	Age (years)	Sex	Age (years)	Sex
10	M	3	M	13	F
3	M	3	M	14	F
2	M	2	F	12	M
4	M	5	F	12	M
3	F				

Abbreviations: F, female; M, male.

**Fig. 4:**
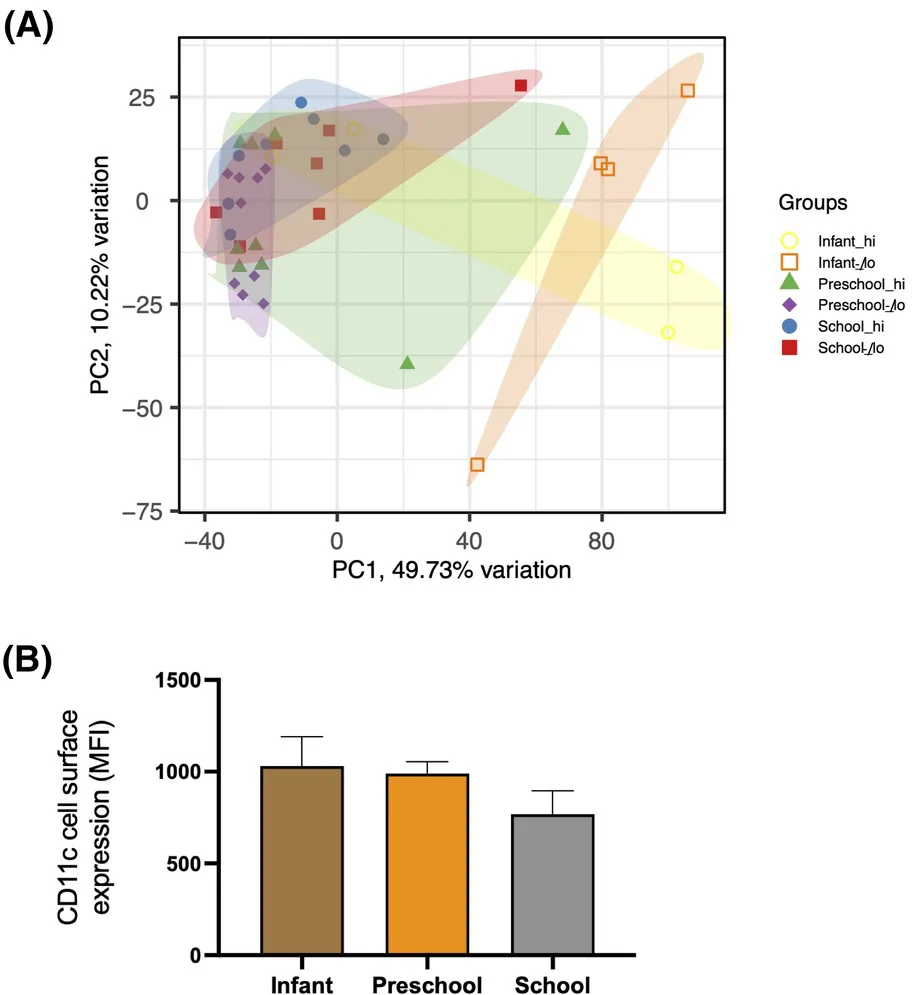
Transcriptomic analysis of Cell surface CD11c^hi^ and CD11c^−/lo^ neutrophils (A) Principal component analysis (PCA). (B) CD11c expression level on neutrophils in three different age groups (infants *n* = 5, preschool *n* = 4, school *n* = 4). One‐way ANOVA with *Bonferroni post hoc* analysis was done. No statistical difference was observed.

Across all age groups, principal component analysis (PCA) revealed no clear separation between CD11c^hi^ and CD11c^−/lo^ neutrophils (Fig. 4A). Principal component 1 (PC1), which explained 49.7% of the total variance, was primarily driven by genes associated with extracellular matrix organization, cell adhesion, and tissue remodeling (COL1A1, COL3A1, COL6A3, FN1, DCN, SPARC, and ANXA2) on the positive axis and by classical neutrophil antimicrobial and granule‐associated genes (MPO, DEFA1/3/4, CEACAM8, CD177, OLFM4, CAMP, and MMP8) on the negative axis (Table S1). Together, these findings indicate that PC1 captures differences in neutrophil functional state rather than clear segregation based solely on CD11c expression. Consistent with this, CD11c expression did not differ significantly across age groups, although a modest decrease in CD11c expression was observed in the school‐age cohort (Fig. 4B).

These findings indicate that cell surface CD11c does not define globally distinct transcriptional states but instead reflects a phenotypic or post‐transcriptional condition. This is consistent with the concept that neutrophil functional heterogeneity is largely governed by post‐transcriptional regulation, granule mobilization and integrin activation dynamics rather than large‐scale transcriptional reprogramming. Importantly, these results suggest that cellular aging/maturation state and chronological (developmental) age represent related but distinct axes of neutrophil heterogeneity, with CD11c expression capturing variation in maturation state.

### Machine learning reveals age‐specific CD11c‐associated molecular programs

**Fig. 5:**
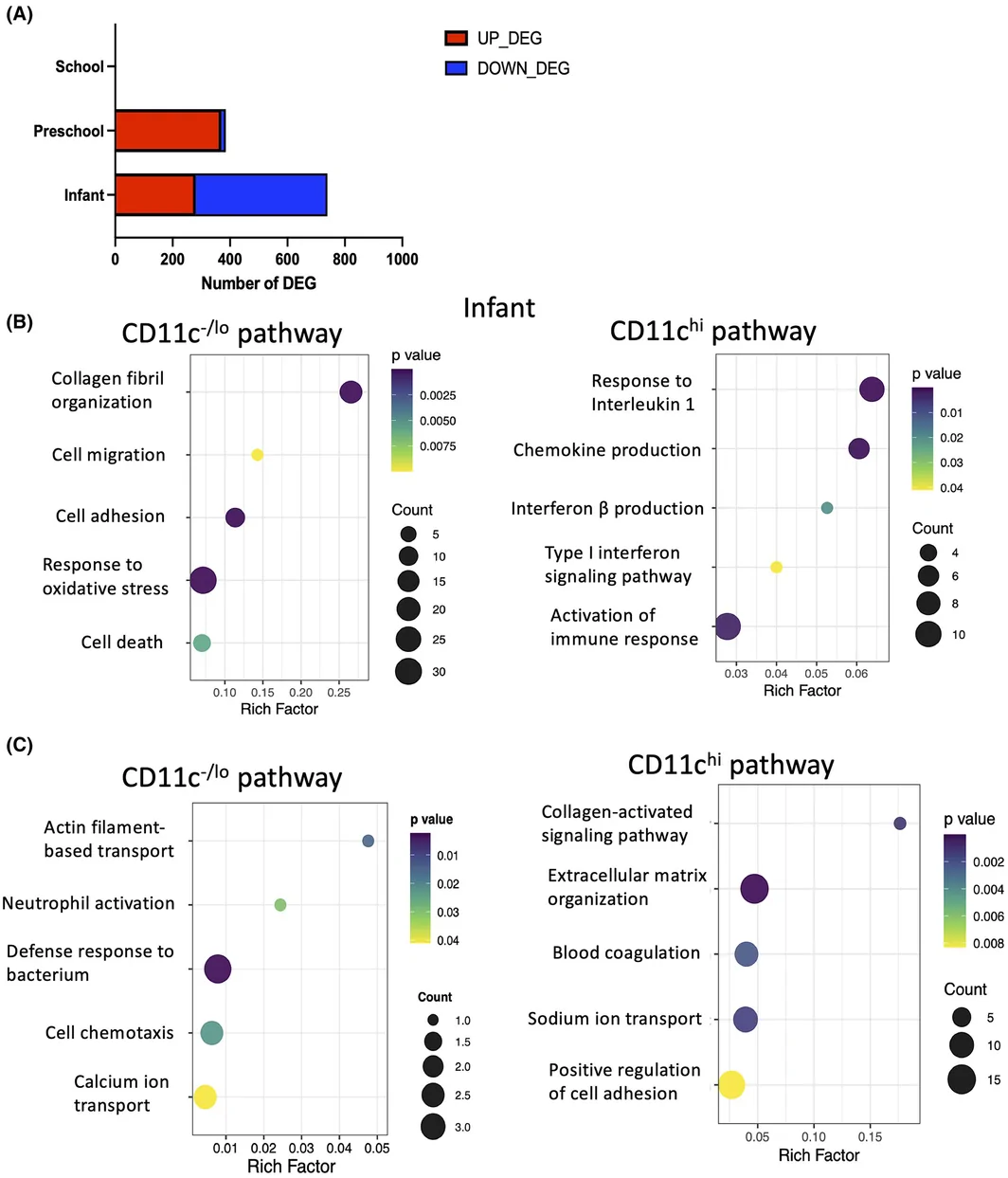
Bulk RNA Sequencing analysis of circulating neutrophils from infants, preschool, and school children. Cell surface CD11c^hi^ and CD11c^−/lo^ neutrophils were sorted and subjected to mRNA transcriptomic analysis following RNA purification, library creation and sequencing. (A) Bar plot depicting differentially expressed genes (DEGs) between CD11c high and CD11c low neutrophils in infants, preschool, and school children. (B, C) Dot plots exhibiting biological pathways associated with the previous DEGs enriched in neutrophils from infants (B) or preschool children (C). P‐value is the false discovery rate.

While we did not see a clear separation between CD11c^hi^ and CD11c^−/lo^ neutrophils, we found age‐dependent presence of DEGs between them. As shown, we have highest DEGs in infants, followed by preschool children (Fig. 5A). School‐aged children had limited DEGs. To further refine the molecular context of CD11c‐associated neutrophil states beyond global transcriptomic comparisons, we performed biological pathway analysis within each developmental group. This analysis identified age‐specific molecular programs associated with CD11c expression, even in the absence of broad transcriptional divergence. Ontology for infants and preschools is shown in Fig. 5B,C.

AI‐based GeneAgent is a recently developed, AI‐driven language agent for gene set analysis [12]. This produces an initial analytic narrative using information based on autonomous database interrogation and self‐verifies the claim. It outperforms the standard GPT‐4 model and also explicitly maps to the underlying dataset to address reproducibility. Using this modality, in infants, CD11c^hi^ neutrophils enriched genes associated with inflammasome activation and inflammatory cell death (CASP4, CASP5, PYCARD), apoptotic priming (BID), immune cell adhesion and signaling (ICAM3), and metabolic and lysosomal regulation (RXRA, TFEB) (Table 2). In contrast, CD11c^−/lo^ neutrophils exhibited enrichment for genes involved in extracellular matrix remodeling (COL1A1, COL1A2, COL3A1, COL5A2, COL6A1, COL6A2, FN1, LUM, DCN, LOX/LOXL family members, MMP2), cellular stress responses (KEAP1, NQO1, heat shock proteins), and integrin‐mediated adhesion pathways (ITGB1, ITGB8, VCAM1, THBS1).

**Table 2:** Biological pathway analysis via AI‐based GeneAgent tool in the infant group.

**CD11c high: apoptosis, metabolism, and cellular communication**
‐ CASP4, CASP5, and PYCARD are involved in inflammasome activation and pyroptosis ‐ BID is a pro‐apoptotic protein that links extrinsic and intrinsic apoptotic pathways ‐ ICAM3 mediates cell adhesion and immune cell trafficking by binding to the leukocyte adhesion ‐ RXRA and TFEB are transcription factors involved in lipid metabolism and lysosomal biogenesis
**CD11c low: Extracellular Region Dynamics and Cellular Stress Response**
‐ COL1A1, COL1A2, COL3A1, COL5A2, COL6A1, COL6A2, FN1, LUM, DCN, LOX, LOXL1, LOXL2, LOXL3, LOXL4, and MMP2 are involved in extracellular matrix remodeling ‐ KEAP1, NQO1, HSP90AB1, HSPB1, HSPB8, and CRYAB are involved in cellular stress response ‐ Integrin signaling and cell adhesion: The genes ITGB1, ITGB8, VCAM1, and THBS1 mediate integrin signaling and cell adhesion, which are critical for cell migration

Likewise, in the preschool group, CD11c^hi^ neutrophils were characterized by transcriptional signatures related to cell–cell adhesion and junctional organization (CDH1, OCLN), protein folding and secretory regulation (AGR2), structural scaffold components (KRT4, KRT19), and extracellular matrix/basement membrane organization (FN1, COL4A1) (Table 3). Of note, epithelial cell genes CDH1 and OCLN were noted in preschool. In contrast, CD11c^−/lo^ neutrophils were enriched for a more classical innate immune effector program, including antimicrobial peptides (CAMP, DEFA1, DEFA1B), lipid signaling pathways (CYP4F3, PNPLA2, OXER1), and calcium‐dependent activation pathways (ATP2A3, CRACR2A). Notably, no significant gene expression differences were identified between CD11c^hi^ and CD11c^−/lo^ neutrophils in the school‐age group, further reinforcing that CD11c does not define a universal transcriptional program across developmental stages.

**Table 3:** Biological pathway analysis via AI‐based GeneAgent tool in preschool group.

**CD11c high: Process: Cell Adhesion and Plasma Membrane Regulation**
‐ CDH1 and OCLN play critical roles in epithelial cell–cell adhesion and tight junction formation. ‐ AGR2 facilitates protein folding within the endoplasmic reticulum, supporting mucin production and epithelial regulation. ‐ KRT4 and KRT19 form intermediate filaments that provide structural stability to epithelial layers. ‐ S100A16 and PERP are associated with cell differentiation and apoptosis. ‐ FN1 and COL4A1 maintain the extracellular matrix and basement membrane
**CD11c low: Innate Immunity and Antimicrobial Activity**
This system integrates antimicrobial activity, lipid signaling, calcium regulation, and immune cell recruitment to support innate immunity and inflammatory responses. The interplay between antimicrobial peptides (CAMP, DEFA1B, DEFA1), lipid metabolism (CYP4F3, PNPLA2, OXER1), and calcium signaling (ATP2A3, CRACR2A) highlights the functional diversity of these proteins in immune defense mechanisms.

### Developmental age establishes the transcriptional and functional baseline of neutrophils

**Fig. 6:**
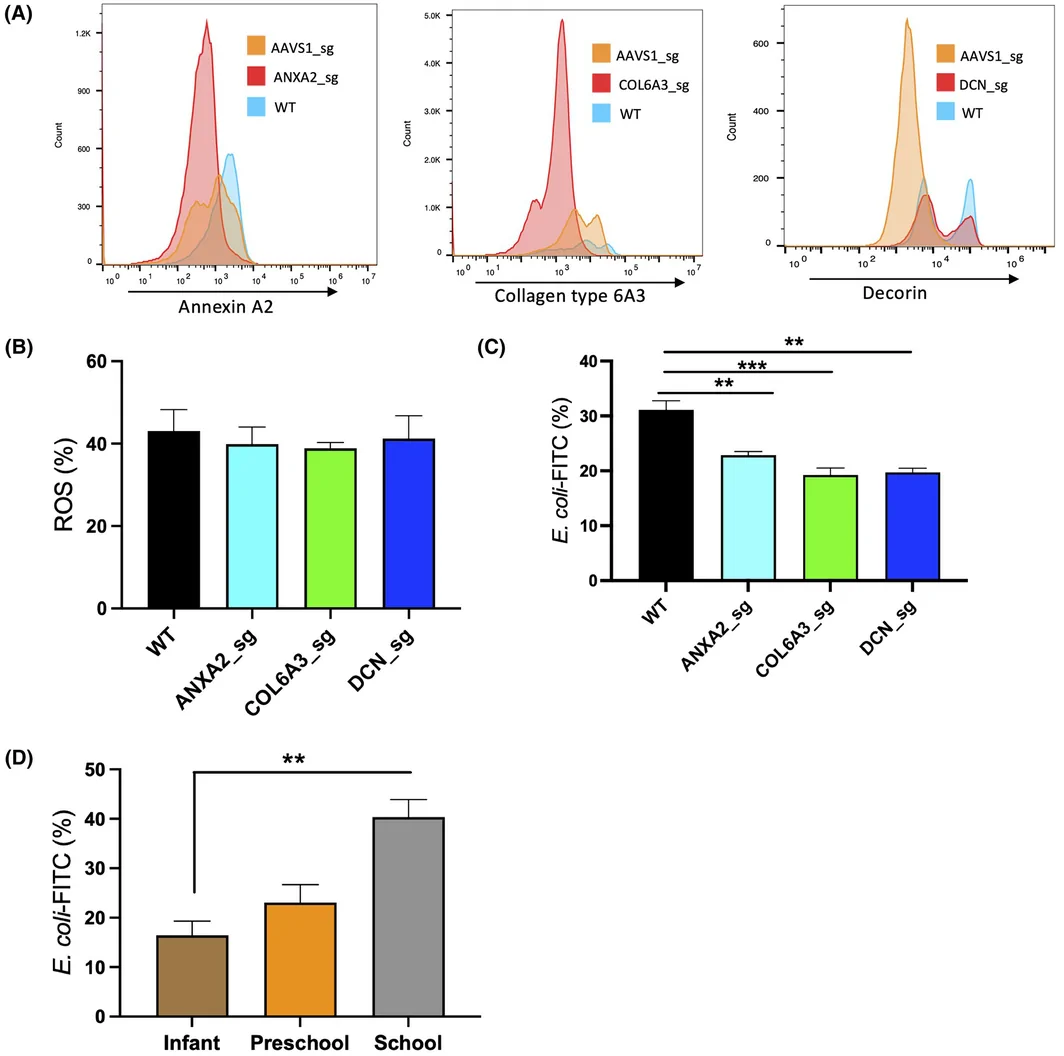
The role of ANXA2, COL6A3, and DCN in differentiated HL‐60 cells. CRISPR/Cas9 deletion of ANXA2, COL6A3, and DCN was performed in HL‐60 cells. (A) We examined the expression level of intracellular Annexin A2, Collagen type 6A3, and decorin in HL‐60 cells following CRISPR/Cas9 deletion using flow cytometry analysis. AAVS1 is a nontarget control. Histogram overlay was shown. (B, C) Reactive oxygen species (ROS) (B) and phagocytosis (C) were tested using them. Data were shown as mean ± S.D. of triplicates. A representative figure of two independent experiments. **P < 0.01. ***P < 0.001. (D) Phagocytosis was tested in three different age groups (infants *n* = 5, preschool *n* = 4, school *n* = 4). Data were shown as mean ± S.D. One‐way ANOVA with *Bonferroni post hoc* analysis was done. **P < 0.01.

Although CD11c‐based stratification did not reveal major transcriptomic differences within age groups, comparative analysis across developmental stages identified significant transcriptional differences between infants and older children. PCA demonstrated a clear separation along the PC2 axis, indicating age‐dependent transcriptional remodeling of neutrophils (Fig. 4A). Because our earlier findings showed that CD11c^hi^ neutrophils exhibit features of aging/maturation (CXCR4^hi^CD62L^lo^) and enhanced phagocytic capacity, we next investigated whether developmental age independently shapes the transcriptional programs that underlie neutrophil functional competence. Genes contributing to the age‐driven separation included ANXA2, COL6A3, and DCN based on PCA analysis, which were selected for further investigation based on their expression patterns and roles in extracellular matrix organization and cellular activation pathways (Table 4). To test their function, we performed CRISPR/Cas9‐mediated deletion in HL‐60 cells and validated the deletion (Fig. 6A). While loss of these genes did not significantly affect ROS production (Fig. 6B), it resulted in a marked reduction in phagocytic capacity (Fig. 6C), indicating that age‐associated transcriptional programs contribute directly to neutrophil‐mediated pathogen clearance. Consistent with these findings, phagocytic capacity was lowest in infant‐derived neutrophils (Fig. 6D), further supporting the conclusion that developmental stage is a primary determinant of neutrophil effector function.

**Table 4:** Differentially expressed genes in infants and their expression in human cell line HL60 cells.

Gene	Name	HL‐60 cell expression	scRNA seq pediatric data
DCN	Decorin	Yes	Yes
CEACAM5	CEA cell adhesion molecule 5	No	No
ANXA2	Annexin A2	Yes	Yes
TNFRSF11B	TNF receptor superfamily member 11b	No	No
COL6A3	Collagen type VI alpha 3 chain	Yes	Yes
SNHG14	Small nucleolar RNA host gene 14	Yes	Yes

**Fig. 7:**
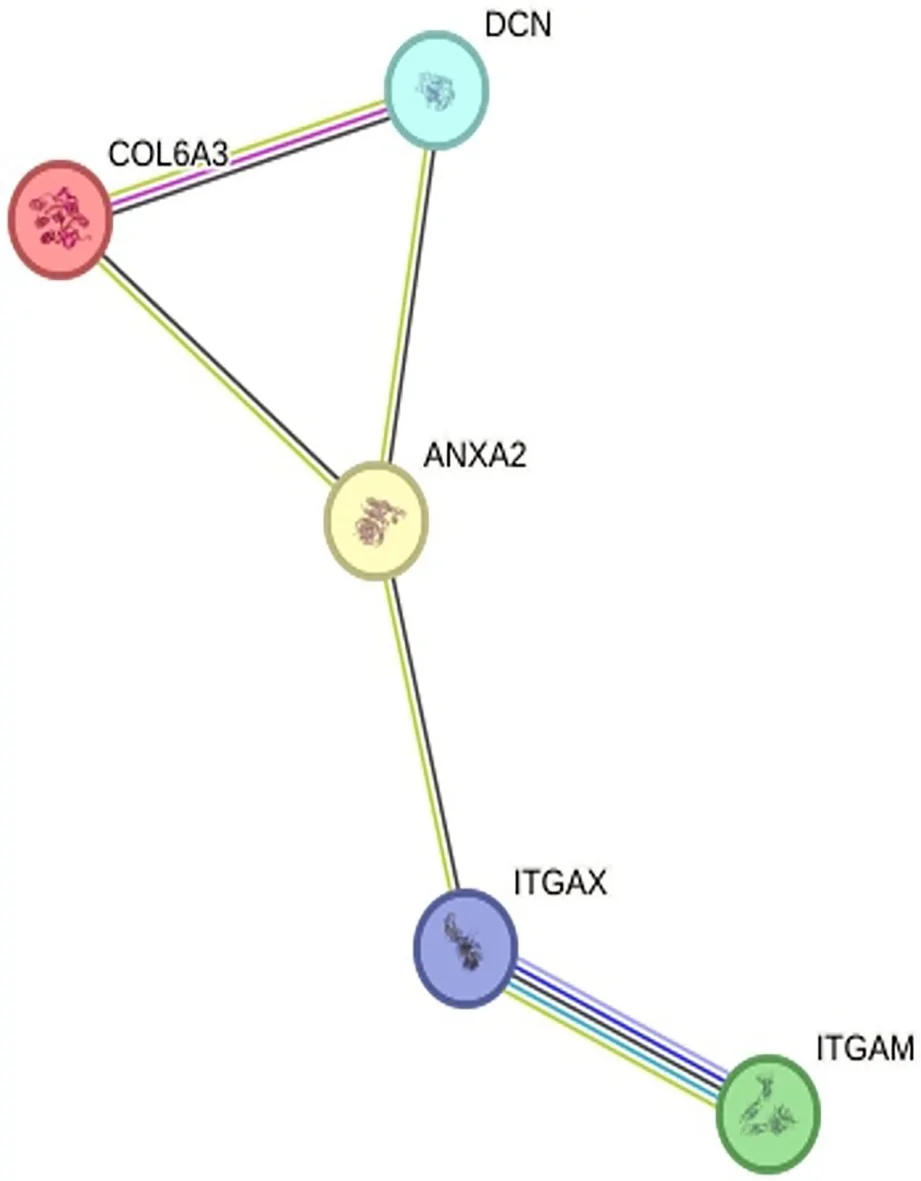
The relationship between ANXA2/COL6A3/DCN and integrin CD11b/CD11c. STRING analysis was performed. *Itgam* is the gene of CD11b and *Itgax* is the gene of CD11c. In the first step, all DEGs were submitted to the STRING PPI database, and a comprehensive interaction network was generated. In the second step, we specifically extracted genes directly connected to ITGAX (CD11c) using a minimum interaction score threshold of 0.4 (medium confidence) to identify its most relevant interaction partners within the DEG set.

CD11c cell surface expression and phagocytosis capability in the three different age groups were in inverse relationship. To further define the molecular context of CD11c‐associated neutrophil states, we performed protein–protein interaction analysis using the STRING database. We focused on CD11c (ITGAX), CD11b (ITGAM), and the identified candidate genes (ANXA2, COL6A3, DCN) tested above using a medium confidence interaction threshold (score ≥0.4). This analysis revealed interconnected networks linking integrins with extracellular matrix components and regulatory proteins involved in cellular adhesion, signaling, and membrane organization (Fig. 7). Given our observations that CD11c expression correlates with CD11b levels and identifies neutrophils with enhanced phagocytic capacity, CD11c likely reflects a cellular state characterized by increased integrin activation and engagement with extracellular ligands. Integrin‐mediated adhesion is tightly coupled to cytoskeletal remodeling and membrane dynamics, processes that are essential for efficient phagocytosis. In addition, molecules such as ANXA2, COL6A3, and DCN, linked to extracellular matrix organization and cell–matrix interactions, may further support these integrin‐dependent functions by facilitating membrane trafficking, adhesion, and phagocytic cup formation.

## Discussion

Here we reported that cell surface CD11c did not delineate transcriptionally very distinct neutrophil subsets within the same developmental stage, but instead identifies a population enriched for features of neutrophil aging. Specifically, CD11c^hi^ neutrophils exhibit phenotypic characteristics consistent with aged cells, including increased CXCR4 expression and reduced CD62L, and are associated with enhanced functional competence, particularly in phagocytosis. Notably, this enhanced functional capacity correlates with increased CD11b expression, a well‐established mediator of neutrophil phagocytic activity, suggesting that CD11c marks a more mature, CD11b^hi^ neutrophil subset rather than acting as a primary functional receptor.

Neutrophil aging is associated with a well‐characterized process of spontaneous granule mobilization and exocytosis [17]. We previously showed that CD11c is predominantly localized within gelatinase (tertiary) granules and to a lesser extent within secretory granules [4]. Neutrophil granules are organized into four classes based on density and content, and their mobilization follows a tightly regulated hierarchical sequence in which secretory vesicles, gelatinase (tertiary) granules, and specific (secondary) granules fuse with the plasma membrane, whereas azurophilic (primary) granules primarily fuse with phagosomal membranes [18]. Delivery of azurophilic granule contents into phagosomes enhances antimicrobial activity, as previously reported [19]. Within this framework, the appearance of CD11c on the cell surface likely reflects granule mobilization associated with neutrophil maturation and aging, rather than *de novo* transcriptional regulation. This interpretation is supported by our transcriptomic analyses, which did not reveal major differences between CD11c^hi^ and CD11c^−/lo^ neutrophils within the same age group, suggesting that CD11c expression is largely governed by post‐transcriptional mechanisms, including granule trafficking and membrane redistribution. While we acknowledge that our small sample size would be a limitation along with sorting asymmetry (~5% for CD11c^hi^ vs ~94% for CD11c^−/lo^), some transcriptomic difference still existed between CD11c^hi^ and CD11c^−/lo^ neutrophils. To further refine the molecular programs associated with CD11c‐defined neutrophil states, we applied a machine learning‐based approach within each developmental group. Very few DEGs were identified between CD11c^hi^ and CD11c^−/lo^ neutrophils in the school‐age cohort. However, the model identified age‐specific molecular programs associated with CD11c expression. In infants, CD11c^hi^ neutrophils were enriched for genes linked to inflammasome activation, apoptotic priming, and metabolic/lysosomal regulation, whereas CD11c^−/lo^ cells were enriched for extracellular matrix remodeling, stress‐response pathways, and integrin‐mediated adhesion. In the preschool group, CD11c^hi^ neutrophils were associated with adhesion‐ and membrane organization programs, while CD11c^−/lo^ cells exhibited a stronger innate immune effector signature, including antimicrobial and inflammatory signaling pathways. These findings further support the concept that CD11c is not a global transcriptional driver but rather associates with context‐dependent molecular programs that vary with developmental stage and converge on pathways relevant to neutrophil maturation and functional readiness.

CD11b, which is structurally homologous to CD11c, also heterodimerizes with CD18 to form a β2 integrin and is highly expressed on neutrophils. CD11b/CD18 binds to iC3b and functions as a well‐established phagocytic receptor. Although CD11c has also been reported to bind iC3b *in vitro*, our data demonstrate that CD11c does not function as a primary phagocytic receptor in neutrophils, as blockade of CD11b completely abrogated phagocytosis. One possible explanation is the relative abundance and avidity of these integrins, as integrin‐mediated adhesion operates through multivalent, cooperative interactions, often described as a ‘Velcro‐like’ mechanism, where cumulative low‐affinity interactions result in high‐avidity binding. Structurally, iC3b engages CD11b and CD11c at distinct sites [22], allowing for potential complementary or cooperative interactions. While CD11c alone does not appear to drive phagocytosis, it may modulate or stabilize integrin‐mediated adhesion or serve as an auxiliary receptor under specific conditions in which CD11b function is limited. It has been reported that CD11c/CD18 on tumor necrosis factor (TNF)‐stimulated neutrophils serves as a fibrinogen receptor by recognizing the α chain of fibrinogen [16], whereas CD11b/CD18 on neutrophils binds to fibrinogen at the γ chain when stimulated with PMA [23]. Here we primarily focused on phagocytosis, ROS formation and NETosis, but underlying the biological implication of neutrophil‐fibrinogen binding can delineate another aspect of cell surface CD11c.

In addition, we explored the difference in neutrophil transcriptomic regulation per age group. Importantly, our findings also reveal a previously underappreciated role of developmental stage in shaping neutrophil transcriptional and functional profiles. We identified significant transcriptomic differences between infant and older pediatric neutrophils, consistent with prior reports demonstrating reduced phagocytic capacity in neonatal neutrophils [24]. As leukocytes undergo progressive maturation after birth [25], these differences likely reflect developmental programming of innate immune responses. In line with this, we observed that phagocytic capacity was lowest in infant‐derived neutrophils, supporting the concept that functional competence increases with maturation. Mechanistically, we identified ANXA2, COL6A3, and DCN as candidate genes associated with age‐dependent transcriptional programs. Functional interrogation using CRISPR/Cas9‐mediated deletion demonstrated that these genes contribute to phagocytic capacity without affecting ROS production, suggesting a specific role in processes such as cell–matrix interactions, membrane organization, and cytoskeletal dynamics. These findings are consistent with the broader concept that efficient phagocytosis requires coordinated regulation of integrin signaling, cytoskeletal remodeling, and extracellular matrix interactions. Developmental age establishes the baseline transcriptional and functional landscape of neutrophils, whereas CD11c identifies a more mature, CD11b^hi^ subset within each developmental stage that is primed for enhanced effector function.

We also acknowledged the limitation of our study. We reported that cell surface CD11c expression was associated with aged neutrophils. However, to further dissect the functional role of CD11c, it may be reasonable to consider sorting on CD11c while controlling for CD11b levels as CD11b and CD11c cell surface expression levels were highly correlated. Second, as mentioned above, we acknowledge the small sample size for bulk RNA seq. This also limits the comparative analysis between sex. Third, we used HL‐60 cells for CRISPR/Cas9 driven gene deletion experiment. While differentiated HL‐60 cells are frequently used as a neutrophil model, they are not necessarily the same with primary neutrophils. For example, they do lack some granule contents.

Taken together, these data support a unifying model in which developmental age determines the baseline transcriptional architecture of neutrophils and influences their functional capacity, particularly phagocytosis. Within each developmental stage, cell surface CD11c identifies a more mature/aged CD11b^hi^ neutrophil subset with enhanced phagocytic competence, but without major global transcriptional divergence from CD11c^−/lo^ cells. The machine learning and network analyses further refine this model by demonstrating that, although CD11c is not a global transcriptional driver, it is associated with distinct, age‐dependent molecular programs that converge on pathways related to adhesion, extracellular matrix interactions, cytoskeletal dynamics, and stress adaptation, all of which are biologically relevant to neutrophil maturation and effector function. Thus, CD11c primarily serves as a marker of neutrophil aging and maturation state, while developmental age shapes the underlying transcriptional and functional landscape upon which CD11c‐associated phenotypes are superimposed. Future research directions include the clarification of the distinct role of CD11b and CD11c such as the delineation of neutrophil–fibrinogen interaction.

## Conflicts of interest

The authors declare no conflict of interest.

## Author contributions

SK did investigation, formal analysis, and wrote an original manuscript. FA performed data curation and edited a manuscript. EM and SK edited a manuscript. MB did investigation. KY performed formal analysis, wrote an original manuscript, and obtained funding.

## Supporting information


**Table S1.** List of gens with loading and absolute loading affecting PC1 axis.

## Data Availability

All data were included in this manuscript. Bulk RNA‐seq data were deposited in GEO (GSE336366).
